# MicroRNA-34a Promotes Ischemia-Induced Cardiomyocytes Apoptosis through Targeting Notch1

**DOI:** 10.1155/2022/1388415

**Published:** 2022-02-28

**Authors:** Jialin Pan, Lili Zhou, Cong Lin, Weihao Xue, Peng Chen, Jiafeng Lin

**Affiliations:** ^1^Division of Cardiology, The Second Affiliated Hospital and Yuying Children's Hospital of Wenzhou Medical University, Wenzhou 325027, China; ^2^Division of Neurology, The Second Affiliated Hospital and Yuying Children's Hospital of Wenzhou Medical University, Wenzhou 325027, China

## Abstract

Myocardial apoptosis occurs during myocardial ischemia. This study aimed to determine the effect of microRNA-34a (miR-34a) in ischemia-induced myocardial apoptosis. Mainly, SD rats were subjected to myocardial ischemia by ligaturing the left anterior descending branch of coronary artery. After rats had myocardial infarction, HE staining and TUNEL staining confirmed a significant increase in apoptosis. The expression of miR-34a was noticeably upregulated, while the expression of Notch1 was downregulated. An increase in caspase-3 and a decrease in Bcl-2/Bax ratio were observed in myocardium. Similar results were observed in the *in vitro* model of cardiomyocyte ischemia and anoxia of this study. When rat cardiomyocytes were administered with serum starvation and microaerophilic system, apoptosis-related proteins were significantly increased. However, transfecting the miR-34a inhibitor into the cardiomyocyte before the serum starvation and hypoxia treatment could increase the ratio of Bcl-2/Bax and downregulate the expression of caspase-3, as well as prevent cardiomyocytes from apoptosis. As opposed to the abovementioned points, the upregulation of miR-34a expression by transfecting miR-34a mimics induced Notch1 reduce and apoptosis-related proteins increase apparently, while upregulation of Notch1 could stimulate apoptosis attributed to miR-34a. Mechanistically, we demonstrated that Notch1 is a direct target of miR-34a. In conclusion, our current results suggested that miR-34a significantly stimulates ischemia-induced cardiomyocytes apoptosis by targeting Notch1.

## 1. Introduction

Coronary heart disease is defined as an acute or chronic heart disease contributing to cardiac ischemia, anoxia, and myocardial necrosis, which results from atherosclerosis-induced coronary arteries stenosis. The morbidity of coronary heart disease increases with the aging of the population that is the leading cause of mortality in the majority of industrialized nations [1, 2]. Numerous studies revealed that the heart, a hypermetabolic organ, has a high demand for blood supply and oxygen, making it more susceptible to ischemia, which may ultimately cause cardiomyocyte apoptosis, necrosis, and subsequent ventricular remodeling [3, 4]. Although coronary catheterization and revascularization have made a remarkable contribution to the outcomes of myocardial infarction (MI), they have also led to a higher number of surviving patients with permanent structural remodeling under the effect of cardiomyocyte apoptosis, necrosis, and fibrosis, thereby frequently leading to heart failure [5]. Thus, novel treatment options that could address the aforementioned problems may be beneficial to manage MI.

Over the last decade, microRNAs (miRs) have been confirmed to be critical for the majority of pathological processes (including apoptosis, proliferation, fibrosis, and metabolism), which shows their therapeutic potential for treating numerous diseases [6]. The miRNA therapy stimulates uncontrolled cardiac repair after MI in pigs, but the dosage should be tightly controlled [7]. miR-34a, one of the three members of the miR-34 family, has been found to be a suppressor of cellular proliferation and promoters of apoptosis, including in cancer cells. miR-34a has been suggested to target a wide variety of genes to regulate diverse functions (such as cell cycle and apoptosis) [8]. miR-34a is highly expressed in the left ventricle of infarcted hearts compared with its expression in other organs, which can serve as a promising strategy to enhance the efficacy of therapies for chronic heart failure [9]. Moreover, miR-34a has been noted to be upregulated over time in cardiomyocytes with cardiac injury and in patients with heart failure [10, 11]. A previous study reported that silencing the entire miR-34 family could protect the heart against pathological cardiac remodeling, as impacted by MI, and improve cardiac functions [12]. However, the effect of miR-34 on myocardial ischemia and its therapeutic potential is largely unclear.

The Notch signaling pathway is taken as an information exchange platform between neighbouring cells [13]. After combining the ligand of Jagged/Delta, it releases the intracellular structure domain into the nucleus, and targets to the recombination signal binding protein for immunoglobulin J kappa transcription factors, subsequently activates the gene transcription of Hes, HRT, etc [14]. A notch signaling pathway is critical to the cell proliferation, apoptosis, and epithelial to mesenchymal transformation [15]. The Notch1 signaling pathway plays an important role in the regulation of apoptosis [16]. Its activation can be protective in myocardial apoptosis [17]. TargetScan analysis showed Notch1 to be one of the targets of miR-34a, which was further validated by luciferase reporter assay in human ovarian cancer cells [18, 19]. Therefore, the miR-34a-Notch1 pathway could be a potential therapeutic target of MI. This study aims to investigate the variation in cardiac miR-34a expression of rats under ischemia and anoxia, as well as to determine the effect exerted by miR-34a on myocardial apoptosis by building an *in vitro* model of cardiomyocyte ischemia and anoxia. Our results revealed the mechanism of miR-34a facilitating myocardial apoptosis in ischemic myocardium, which suggests the therapeutic potential of miR-34a for myocardial ischemia.

## 2. Materials and Methods

### 2.1. Experimental Animals and Treatments

Male Sprague-Dawley rats (weighing 250–300 g) were provided by the Chinese Academy of Sciences. Rats were treated according to the Guide for the Care and Use of Laboratory Animals by the National Institutes of Health (NIH). The rats were randomized into three groups: blank control (BC) group (without any intervention), MI model group (ligation of the left anterior descending coronary artery), and sham operation (negative control; NC) group (*n* = 8 each group). Rats were administered with general anesthesia (2% Isoflurane/O2) before performing a coronary artery occlusion surgery. Successful ligation of the left anterior descending coronary artery was demonstrated by ST-segment elevation in lead II (>0.2 mV) in the postoperative electrocardiogram (ECG) compared with preoperative ECG. The animal protocols conducted in this study, which were approved by the Animal Welfare Committee of Wenzhou Medical University (Number: wydw2014-0058), followed national and institutional regulations, and were compliant with the Guide for the Care and Use of Laboratory Animals published by National Institutes of Health.

### 2.2. Cell Lines and Culture Conditions

The rat cardiomyocyte line (H9C2) was obtained from The Cell Bank of Type Culture Collection of the Chinese Academy of Sciences. H9C2 cells were maintained in DMEM (Gibco; Thermo Fisher Scientific, Inc.) supplemented with 10% fetal calf serum (Gibco; Thermo Fisher Scientific, Inc.) at 37°C in an incubator with 5% CO_2_. The medium was replaced every 2–3 days, and the cells were sub-cultured or subjected to experimental procedures at 80–90% confluency. To induce myocardial ischemia and hypoxia, H9C2 cells were cultured in serum-free medium (starvation group) at 37°C in an incubator with 5% CO_2_, 94% N_2_, and 1% O_2_ (microaerophilic system with an anaerobic gas mix) for 12 h (starvation treatment). In the normal group, H9C2 cells were cultured in 10% fetal calf serum at 37°C in an incubator with 5% CO2.(We followed the methods of Jialin Pan, Yongjian Geng et al. 2014 [20].

### 2.3. Cell Transfection

When H9C2 cells reached ∼80% confluence, they were transfected with 10 pmol miR-34a mimic (5′-UGGCAGUGUCUUAGCUGGUUGU-3′) (Shanghai GenePharma Co., Ltd.), miRs mimic negative control (miR-NC), 10 pmol miR-34a inhibitor (miR-inhibitor) (5′-ACCGUCACAGAAUCGACCAACA-3′) (Shanghai GenePharma Co, Ltd.), and 2 mg pcDNA-Notch1 (Taijin Saier Biotechnology) using Lipofectamine^®^ 2000 (Invitrogen; Thermo Fisher Scientific, Inc.) following the manufacturer's instructions. The concentration of FAM-miRNA was 10pmol/l, and the concentration of pcDNA-Notch1 was 2 mg/l. Transfection of miR-34a inhibitor was conducted 24 h before starvation treatment. We followed the methods of Jialin Pan, Yongjian Geng et al. 2014 [20].

### 2.4. The qRT-PCR Analysis

Total RNAs from rat myocardium tissues (weight, 30 mg) and H9C2 cells were extracted with TRIzol^®^ (Invitrogen; Thermo Fisher Scientific, Inc.) and then leached with chloroform and concentrated with isopropyl alcohol. The purity of total RNA was measured with a spectrophotometer (DU800; Beckman Coulter, Inc.), and its quality was determined by formaldehyde denaturing gel electrophoresis. miRNAs were isolated and then purified using a *miRNA Isolation Kit* (Ambion; Thermo Fisher Scientific, Inc.). According to the manufacturer's instructions, cDNA was reverse transcribed from RNA using a *MiScript II RT Kit* (Qiagen, Inc.). miRNA-specific qRT-PCR primers were applied in qRT-PCR together with the miScript Universal primer included in the aforementioned kit. The sequence of the rno-miR-34a-specific primer was 5′-TGCGCTGGCAGTGTCTTAGCTG-3′, and that of the U6 primer was 5′-CAAGGATGACACGCAAATTCG-3'. The sequences of the Notch1-specific primers were forward, 5′-CCGCTGTGAGTCGGTCATTA-3′, and reverse, 5′-GGCACCTACAGATGAATCCA-3'. PCR was performed in an ABI 7500 Fast Real-Time PCR system (Applied Biosystems; Thermo Fisher Scientific, Inc.) using a PCR kit (SYBR Green Realtime PCR Master Mix). The PCR thermocycling conditions were as follows: 40 cycles, involving 95°C for 5 sec and 60°C for 34 sec. The relative expression of miR-34a was normalized to that of U6 RNA, and the ΔΔCq method was adopted to determine the relative expression of the sample gene as follows: ΔCq sample = Cq sample − Cq U6 sample; ΔCq control = Cq control − Cq U6 control; ΔΔCq = ΔCq sample − ΔCq control, where Cq indicates the number of cycles required by the fluorescence signal intensity to reach the threshold value in the PCR amplification process. The experiment was conducted in triplicate. We followed the methods of Jialin Pan, Yongjian Geng et al. 2014 [20].

### 2.5. Western Blotting Assay

Myocardial tissues (weighing 50 mg) were lysed in a lysis buffer (RIPA : PMSF, 99 : 1; Beyotime Institute of Biotechnology). Protein concentration was measured using a BCA protein assay (Pierce; Thermo Fisher Scientific, Inc.). The protein samples (50 *μ*g/lane) were separated by SDS-PAGE (Beyotime Institute of Biotechnology) and subsequently transferred to polyvinylidene fluoride membranes. The membranes were blocked for 1.5 h at room temperature with 5% skimmed milk, followed by incubation overnight at 4°C with each primary antibody (all from Abcam). Upon three washes with TBS-Tween (TBST) buffer, the membranes were then incubated with a secondary antibody (goat anti-rabbit IgG at 1 : 4,000 dilution) for 1.5 h at room temperature and then washed three times with TBST buffer. The blots were analyzed using the Odyssey Infrared Imaging system (LI-COR Biosciences). The relative quantity of protein on each blot was normalized to that of *β*-actin, and semi-quantitative analysis of the results was conducted using the AlphaEaseFC Imaging System (ProteinSimple). The experiment was repeated ≥3 times. We followed the methods of Jialin Pan, Yongjian Geng et al. 2014 [20].

### 2.6. TUNEL Staining

4 h after surgery, the hearts of rats were rapidly excised and sectioned into 5 *μ*m thick sections. The sections baked in an oven at 60°C for 30 min, dewaxed with xylene (5 min × 3 times), and dehydrated with 100%, 95% and 70% ethanol, respectively, with each dehydration 3 times. Then, the sections were incubated with protein kinase K for half an hour, washed with phosphate‐buffered solution (PBS), and added the TdT and Luciferase-labeled dUTP with reaction for 1 h at 37°C. The TUNEL mix contained 450 *μ*L label solution and 50 *μ*L enzyme solution. The sections of myocardial tissues were incubated with 50 *μ*L TUNEL mix at 37°C for 1 hour. The sections were washed in phosphate‐buffered solution (PBS) thrice and stained with DAPI and *α*‐actin. The sections were observed by fluorescence microscopy.

### 2.7. Haematoxylin‐Eosin (HE) Staining

4 h after surgery, the left ventricle of hearts was rapidly excised and put in 10% formaldehyde solution, dehydrated in ethanol gradient, embedded in paraffin, and cut down into slices of 5 *μ*m. After deparaffinage, the samples were stained with haematoxylin and eosin. Then, the slices were mounted and observed under a light microscope.

### 2.8. Target Identification and Dual Luciferase Assay

Notch1, as predicted by bioinformatics analysis with TargetScan (http://www.targetscan.org), is a potential target of miR-34a, which was verified using a dual reporter luciferase assay. To incorporate the 3′ untranslated region (UTR) of Notch1 into the multiple cloning sites of the pGL3-REPORT luciferase vector (Promega Corporation), two steps of cloning strategy were performed, i.e., ligation of the 3′UTR PCR product into the vector, and introduction of novel restriction sites on the PCR product, which was then subcloned into the pGL3-REPORT vector.

H9C2 cells were transfected with miR-34a mimics, as well as with wild-type (WT) or mutant (MUT) pGL3-Notch1. The concentration of miR-34a mimics was 10 pmol/l, while the concentration of vector was 2 mg/l. Luciferase activity was determined using a luciferase reporter kit (Promega Corporation). The experiment was conducted according to the instructions of the luciferase reporter kit (Promega Corporation). *Renilla* luciferase activity was used for normalization.

### 2.9. Statistical Analysis

The data were analyzed with SPSS 17.0 software (SPSS, Inc.). All experiments were performed in triplicate, and the results are expressed as the mean ± SD. The differences between multiple groups were analyzed using a one-way ANOVA followed by a Tukey's post hoc test. *p* < 0.05 was considered to indicate a statistically significant difference.

## 3. Results

### 3.1. MI Upregulated miR-34a Expression and Promoted Apoptosis

**Figure 1:**
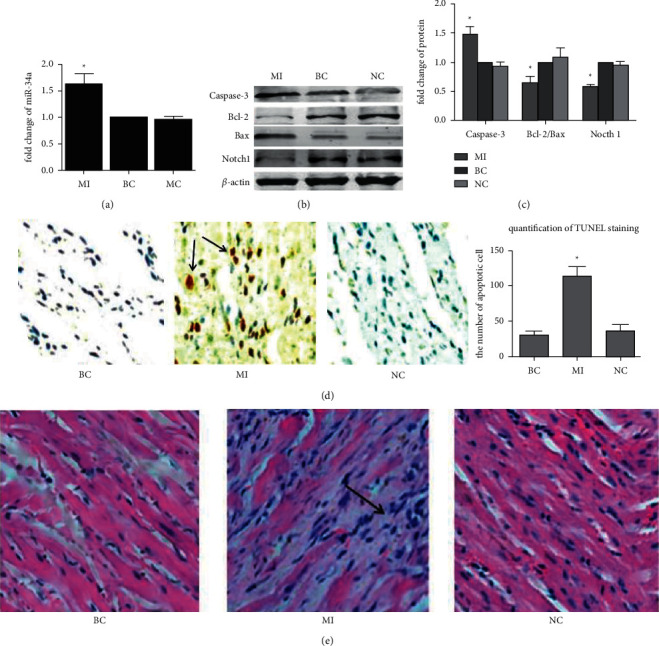
MI upregulated miR-34a expression and promoted apoptosis. MI: myocardial infarction group (myocardial infarction model); BC: blank control group (normal); NC: negative control group (sham operation). (a) qRT-PCR is showing the expression of miR-34a in the myocardium of rats. (b) Expression of caspase-3, Bcl-2, Bax, and Notch1 in different groups detected by performing the western blotting assay. (c) The quantified results of the western blotting assay. (d) The results of TUNEL staining. MI: it is area of myocardial infarction, and there are a large number of apoptotic cardiomyocytes with different shades of brown cell nucleus (as indicated by the arrow). BC and NC: they are noninfarct area, and the cardiomyocytes without brown cell nucleus are arranged orderly. (e) The results of HE staining. MI shows few blood vessels and rarely viable myocardium with irregular cell structure and sequence in the infarct area (as indicated by the arrow). BC and NC show that normal cardiomyocytes in the noninfarct area are orderly (^*∗*^*p* < 0.01 vs NC).

As revealed from the results of qRT-PCR analysis, the rats in the MI group had markedly higher miR-34a expression than those in the BC and NC groups (*p* < 0.01) (Figure 1(a)). After MI, the miR-34a expression was significantly upregulated in the myocardium. Caspase-3 and the ratio of Bcl-2/Bax are important markers of apoptosis. As revealed by western blotting, caspase-3 was markedly increased, while Notch1 and the ratio of Bcl-2/Bax were noticeably decreased in the MI group compared with those in the BC and NC groups (*p* < 0.01) (Figures 1(b) and 1(c)). HE staining and TUNEL staining also confirmed a significant increase in apoptosis (Figures 1(d) and 1(e)). These results indicated that the miR-34a expression and apoptotic cardiomyocytes were significantly increased after MI.

### 3.2. Starvation Treatment Promoted Apoptosis, While Downregulation of miR-34a Inhibited This Effect

**Figure 2:**
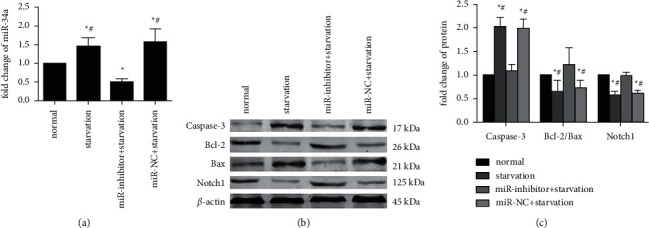
Starvation treatment upregulated miR-34a expression and promoted apoptosis, while downregulation of miR-34a prevented cardiomyocytes from apoptosis attributed to starvation treatment. (a) qRT-PCR is showing the expression of miR-34a in H9C2 cells. (b) Expression of caspase-3, Bcl-2, Bax, and Notch1 in different groups detected by performing the western blotting assay. (c) The quantified results of the western blotting assay (^*∗*^*p* < 0.01 vs normal, ^#^*p* < 0.01 vs miR-inhibitor + starvation).

To induce myocardial ischemia, H9C2 cells were cultured in a serum-free medium and microaerophilic system *in vitro*. The miR-34a expression was significantly upregulated and the apoptotic cardiomyocytes were significantly increased. After starvation treatment, qRT-PCR analysis revealed that cells with starvation treatment had a markedly higher level of miR-34a expression (*p* < 0.01) (Figure 2(a)). The results of Western blotting indicated that Notch1 expression and the Bcl-2/Bax ratio were markedly decreased, while caspase-3 expression was significantly upregulated when H9C2 cells were subjected to starvation treatment (*p* < 0.01) (Figures 2(b) and 2(c)).

Myocardial ischemia promoted apoptosis and upregulated miR-34a which is associated with apoptosis, which suggests that myocardial ischemia might promote apoptosis by upregulating miR-34a. Therefore, we further designed experiments to prove apoptosis induced by myocardial ischemia could be alleviated by downregulation of miR-34a expression. Transfection with a miR-34a inhibitor markedly downregulated miR-34a expression in H9C2 cells (*p* < 0.01), suggesting that the miR-34a inhibitor successfully transfected and repressed miR-34a expression (Figure 2(a)). Starvation treatment promoted H9C2 cells apoptosis. However, when H9C2 cells were transfected with the miR-34a inhibitor, apoptosis induced by starvation treatment was prevented. Western blotting indicated that Notch1 and the Bcl-2/Bax ratio were significantly higher, while caspase-3 expression was lower in the miR-inhibitor group compared with those in the miR-NC and starvation groups (*p* < 0.01; Figures 2(b) and 2(c)).

### 3.3. miR-34a Promoted Apoptosis and Downregulated Notch1 Expression

**Figure 3:**
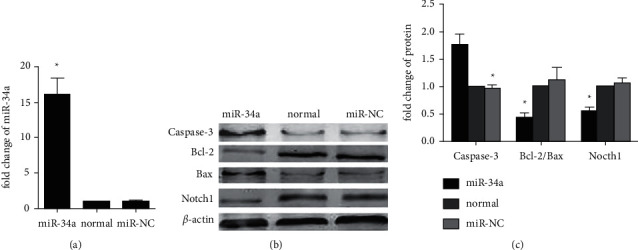
miR-34a promoted apoptosis and upregulated Notch1 expression. (a) qRT-PCR is showing the expression of miR-34a in H9C2 cells. (b) Expression of caspase-3, Bcl-2, Bax, and Notch1 in different groups detected by performing western blotting assay. (c) The quantified results of western blotting assay (^*∗*^*p* < 0.01 vs normal).

After miR-34a mimic transfection, qRT-PCR analysis verified that H9C2 cells had a significantly higher level of miR-34a expression (by ∼16-fold) relative to that of the miR-NC group (*p* < 0.01), indicating that the miR-34a mimic was successfully transfected into H9C2 cells and increased miR-34a expression (Figure 3(a)). Activation of the Notch1 signaling pathway has a protective effect on apoptosis. After miR-34a mimic transfection, Notch1 expression was reduced and apoptosis was enhanced in H9C2 cells. Western blotting indicated that Notch1 expression and the ratio of Bcl-2/Bax were strongly decreased, while caspase-3 expression was markedly upregulated in the miR-34a group compared with those in the miR-NC and normal groups (*p* < 0.01; Figures 3(b) and 3(c)). These results suggested that Notch1 played an important role in miR-34a promoting cardiomyocytes apoptosis.

### 3.4. Upregulation of Notch1 Rescued Apoptosis Attributed to miR-34a

**Figure 4:**
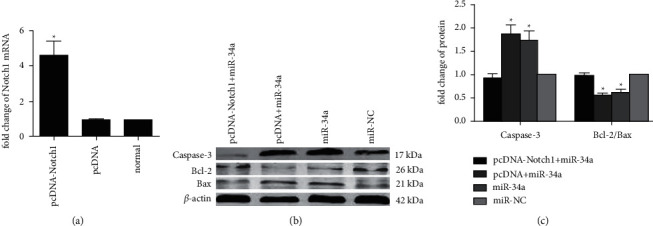
Upregulation of Notch1 rescued apoptosis attributed to miR-34a. (a) qRT-PCR is showing the mRNA expression of Notch1 in H9C2 cells. (b) Expression of caspase-3, Bcl-2, and Bax in different groups detected by performing the western blotting assay. (c) The quantified results of the western blotting assay (^*∗*^*p* < 0.01 vs normal, #*p* < 0.01 vs miR-inhibitor + starvation).

The expression of Notch1 was upregulated by transfecting with pcDNA-Notch1. After transfection of pcDNA-Notch1, qRT-PCR analysis verified that H9C2 cells had a significantly higher expression level of Notch1 compared with that of the empty pcDNA vector group (*p* < 0.01; Figure 4(a)), indicating that pcDNA-Notch1 was successfully transfected into H9C2 cells and upregulated Notch1 expression. As aforementioned, the transfection of miR-34a mimic in H9C2 cells promoted apoptosis, whereas Notch1 overexpression could partially rescue miR-34a-induced apoptosis. Western blotting assay demonstrated that caspase-3 expression was markedly decreased, while the ratio of Bcl-2/Bax was strongly increased in the pcDNA-Notch1 + miR-34a group compared with those in the miR-34a group (*p* < 0.01; Figures 4(b) and 4(c)).

### 3.5. miR-34a Targeted Notch1

**Figure 5:**
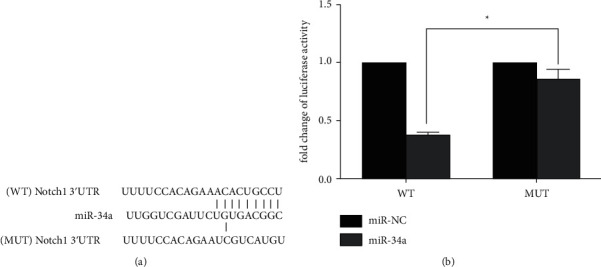
miR-34a targeted Notch1 in H9C2 cells. WT: wild-type PGL3-Notch1; MUT: the mutated PGL3-Notch1. (a) TargetScan analysis of miR-34a showing Notch1 as the potential target of miR-34a. (b) Dual luciferase assay. Luciferase activity in WT group significantly decreased compared with that of MUT group (^*∗*^*p* < 0.01).

Notch1 could be one of potential targets of miR-34a, as predicted by bioinformatic analysis using TargetScan. To confirm whether the 3′UTR of Notch1 is a functional target of miR-34a, a reporter plasmid containing the 3′UTR of Notch1 (WT or MUT) was cloned between the firefly luciferase reporter gene and the polyA tail. As suggested by the luciferase assay results, co-transfection of miR-34a and pGL3-Notch1-WT resulted in a significantly reduced luciferase activity compared with that of the MUT group (*p* < 0.01; Figure 5). This result proved that Notch1 was a target gene of miR-34a.

## 4. Discussion

The heart is a hypermetabolic organ with a high demand for blood and oxygen. In coronary heart diseases, persistent ischemia contributes to cardiomyocyte apoptosis and necrosis. Acute MI is recognized as a common cardiovascular event that causes cardiac remodeling and consequently leads to chronic heart failure. Several miRs have been shown to control important processes that contribute to the pathophysiological consequences of acute MI [21]. miR-34a belongs to one of several evolutionarily conserved families of miRNAs, namely, miR-34 [22]. The miR-34 family are expresses in almost all vertebrates, and in almost every tissue but are scarcely expressed in lung tissue [23]. The miR-34a has been demonstrated to play an important role in the apoptosis function of tumor progression, particularly during the regulation of tumorigenesis and development [24, 25]. Knockdown of miR-34 contributed to breast cancer cell proliferation and apoptosis, whereas the overexpression of miR-34a prevented the development of cancer [26]. miR-34a has been found to be highly expressed in cardiac tissue, and loss of miR-34a improves cardiac function and reduces cell death in aging hearts. Serum miR-34a levels in acute myocardial infarction (AMI) patients and rats were significantly higher than healthy subjects and sham rats, which could promote cardiomyocyte apoptosis via negatively regulating ALDH2 [27]. In addition, acute an antagomir of miR-34a delivered intravenously within several hours post-MI also significantly improved cardiac function at two weeks [11]. miR-34a plays pro-apoptotic and pro-senescence roles in mesenchymal stem cell by targeting SIRT1; furthermore, inhibition of miR-34a might have important therapeutic implications in MSC-based therapy for myocardial infarction [28]. In another study, circulating miR-34a upregulation was detected after acute MI, which could be a useful biomarker for predicting left ventricular remodeling, and the miR-34a level is associated with increased risk of mortality or heart failure [29]. In this study, we found that miR-34a was aberrantly upregulated after acute MI *in vivo*. The same results were observed in cardiomyocytes were subjected to a serum-free medium and microaerophilic system to mimic an ischemic and hypoxia environment *in vitro*.

To further assess the mechanism by which miR-34a regulates cardiomyocyte apoptosis, we specifically examined the expression of three apoptosis-related genes, including caspase-3, Bcl-2, and Bax. As is known to all, the process of cell apoptosis includes at least two crucial phases: The effective phase, which depends on the Bcl-2 family, and the degradation phase, which depends on caspases. At the effective phase, cytochrome c is released from mitochondria, which is modulated by Bcl-2/Bax. However, at the degradation phase, caspase-3 acts as the final executor in apoptotic events. For this reason, the activity of caspase-3 and the ratio of Bcl-2/Bax can effectively reveal the extent of apoptosis [30–32]. Anti-apoptotic effects of Bcl-2 and pro-apoptotic effects of Bax and caspase-3 are very common in many organs, including the heart [33]. Through bidirectional regulation of miR-34a expression *in vitro,* our results disclosed the regulatory effect of miR-34a on the apoptosis of myocardial cells under serum starvation and microaerophilic system to mimic an ischemic environment. Our data suggest that miR-34a regulates the expression of apoptosis indicators, including caspase-3, Bcl-2, and Bax, and promotes apoptosis. Upregulation of miR-34a expression has also been reported in doxorubicin-induced cardiotoxicity, and silencing miR-34a could exert a cardioprotective effect in doxorubicin toxicity [34, 35]. Upregulation of miR-34a was observed both in the serum of patients with acute MI and in rats subjected to MI, which was further demonstrated to promote apoptosis [10, 27]. In another study, cardiac miR-34a expression was augmented in aged mice, leading to a markedly elevated level of myocardial apoptosis [11]. As suggested by the aforementioned findings, ischemic cardiomyocytes overexpress miR-34a, which is associated with cardiomyocyte apoptosis. The potential cardioprotective effect of miR-34a-knockdown was shown in another study that transfection with a miR-34a antagomir inhibited myocardial cell apoptosis after infarction via the Wnt/*β*-catenin signaling pathway [36]. miR-34a was also found to be upregulated by high glucose-treated cardiomyocytes, and the Bcl-2 gene was identified as one of the targets of miR-34a. miR-34a mimics inhibited Bcl-2 expression and stimulated cardiomyocyte apoptosis [37]. In another study on stem cell therapy for ischemic heart disease, miR-34a repressed heat shock protein 70 expression, which protected Sca-1 stem cells from apoptosis, and knockdown of miR-34a improved Sca-1 stem cell survival [38].

Notch1 was predicted to be a potential target of miR-34a based on miRs target prediction software (TargetScan, PicTar, and miRBase). The Notch1 signaling pathway is an apoptosis-related pathway that is involved in the apoptosis of various cell types [39–41]. Notch1 acts as an endogenous myocardial protective factor through the RISK/SAFE/HIF-1 alpha signaling, which reduces myocardial intracellular reactive oxygen species (ROS), enhances the myocardiocytes vitality, and significantly reduces the myocardial ischemia reperfusion injury [42]. On the other hand, Notch1 signaling was reported to regulate the expression of key proteins of mitochondrial oxidative phosphorylation and modulate the dynamic balance of mitochondrial fusion/fission via RBP-Jk dependent transcriptional activation of Mfn1 and Drp1 in myocardial cells [43]. Additionally, Notch1 signaling could inhibit apoptosis because the constitutive overexpression of the intracellular domain of Notch1 promoted proliferation and suppressed apoptosis by inhibiting cytoplasmic mitochondrial membrane depolarization, cytochrome *c* release, and activation of caspase-9 and caspase-3. The survival-promoting effect of Notch1 was also accomplished by upregulation of the anti-apoptotic proteins Bcl-2, downregulation of the pro-apoptotic proteins Bax, and blockade of Bax multimerization [44]. Blocking the Notch1 signaling pathway stimulated hypoxia/reoxygenation-induced cardiomyocyte apoptosis, whereas the overexpression of intracellular Notch1 had a cardioprotective effect [45]. Inhibition of miR-34a-5p could protect myocardial ischemia reperfusion injury-induced apoptosis and attenuate the accumulation of reactive oxygen species through the regulation of Notch1 signaling [46]. Our findings are consistent with this report. In this study, we found that Notch1 expression reduced after MI and was negatively regulated by miR-34a. Furthermore, Notch1 overexpression was found to partly reverse the effects of miR-34a overexpression on cardiomyocyte apoptosis, and the dual luciferase assay further validated Notch1 was a target of miR-34a. Such a finding was also confirmed in another study, which revealed that miR-34 hinders the proliferation of human ovarian cancer cells by inducing autophagy and apoptosis, and inhibits cell invasion by targeting Notch1 [19].

In conclusion, we found increased expression of miR-34a in myocardium after myocardial infarction, and verified that miR-34a was closely associated with myocardial apoptosis *in vitro*. In addition, Notch1 was identified as a target of miR-34a. These results suggested that ischemia induces the upregulation of miR-34a expression, thereby facilitating ischemia-induced cardiomyocytes apoptosis by targeting Notch1. Our findings may provide a novel thought for the study of pathogenesis and treatment of coronary heart diseases. However, the limitation of this study is the serum starvation and microaerophilic system treatment cannot completely simulate *in vitro* model for myocardial ischemia. Unfortunately, no better *in vitro* model of myocardial ischemia and hypoxia has been reported. In addition, our work still lacks *in vivo* studies about the specific mechanism of miR-34a/Notch1 signaling pathways in myocardial apoptosis, but that will be our future work.

## Data Availability

The datasets used and/or analyzed during the current study are available from the corresponding author on reasonable request.
